# Towards Higher Electric Conductivity and Wider Phase Stability Range via Nanostructured Glass-Ceramics Processing

**DOI:** 10.3390/nano11051321

**Published:** 2021-05-17

**Authors:** Tomasz K. Pietrzak, Marek Wasiucionek, Jerzy E. Garbarczyk

**Affiliations:** Faculty of Physics, Warsaw University of Technology, Koszykowa 75, 00-662 Warsaw, Poland; tomasz.pietrzak@pw.edu.pl (T.K.P.); marek.wasiucionek@pw.edu.pl (M.W.)

**Keywords:** glass-ceramics, nanocomposites, nanomaterials, nanocrystallization, conducting glasses, electrode materials, electrolyte materials

## Abstract

This review article presents recent studies on nanostructured glass-ceramic materials with substantially improved electrical (ionic or electronic) conductivity or with an extended temperature stability range of highly conducting high-temperature crystalline phases. Such materials were synthesized by the thermal nanocrystallization of selected electrically conducting oxide glasses. Various nanostructured systems have been described, including glass-ceramics based on ion conductive glasses (silver iodate and bismuth oxide ones) and electronic conductive glasses (vanadate-phosphate and olivine-like ones). Most systems under consideration have been studied with the practical aim of using them as electrode or solid electrolyte materials for rechargeable Li-ion, Na-ion, all-solid batteries, or solid oxide fuel cells. It has been shown that the conductivity enhancement of glass-ceramics is closely correlated with their dual microstructure, consisting of nanocrystallites (5–100 nm) confined in the glassy matrix. The disordered interfacial regions in those materials form “easy conduction” paths. It has also been shown that the glassy matrices may be a suitable environment for phases, which in bulk form are stable at high temperatures, and may exist when confined in nanograins embedded in the glassy matrix even at room temperature. Many complementary experimental techniques probing the electrical conductivity, long- and short-range structure, microstructure at the nanometer scale, or thermal transitions have been used to characterize the glass-ceramic systems under consideration. Their results have helped to explain the correlations between the microstructure and the properties of these systems.

## 1. Introduction

Today’s world depends on technology much more than at any time in the past. One of the main foundations of modern technology are novel, advanced materials, whose properties should be “tailored on-demand” to meet the requirements of given applications. Designing such materials and introducing them to the market is a great challenge but also a major driving force for modern solid-state physics, chemistry, and materials science and engineering. Over the past few decades, in contrast to previous periods, there has been a growing understanding that the physicochemical properties of materials depend not only on their chemical composition or crystallographic structure but also on their microstructure, especially at a nanometer scale. Abundant examples from the history of nanostructured materials have confirmed that the nanoscopic heterogeneity of materials or the nanoscopic character of their elementary building blocks (roughly 1–100 nm in size) may lead to substantial changes in the materials’ properties: mechanical, electrical, magnetic, optical, thermal, catalytic, and many others [1,2]. Some of these changes can be advantageous from the viewpoint of applications. Therefore, many research groups have been motivated to synthesize new nanostructured materials and study their structure-properties correlations, focusing on these correlations when the size of elementary components of the material (grains, layer, wires) is at the nanometer scale. 

One of the efficient routes to nanostructured materials is the thermal nanocrystallization of glasses. The method consists of annealing glasses to obtain nanocrystalline or crystalline-amorphous materials with the required microstructure. In that processing, the initial glassy material turns into a glass-ceramic one. The recently updated definition of glass-ceramics states: “Glass-ceramics are inorganic, non-metallic materials prepared by controlled crystallization of glasses via different processing methods. They contain at least one type of functional crystalline phase and a residual glass. The volume fraction crystallized may vary from ppm to almost 100%” [3].

Practical advantages of glass-ceramic processing include relative technological simplicity, scalability, reproducibility, and versatility. Among the main advantages of glass-ceramic materials are their dense, non-porous microstructure, good mechanical, electrical, thermal, and magnetic properties, or optical transparency. Such materials have already found many practical applications, e.g., as fiber optic devices, heat-resistant materials (Li_2_O–Al_2_O_3_–SiO_2_), hard disk substrates (Li_2_O–SiO_2_), dental materials (K_2_O–Al_2_O_3_–SiO_2_), and many others [4,5,6]. 

It has been recognized that nanocrystallization of certain ionically- or electronically conducting glasses may produce glass-ceramic materials with substantially enhanced electrical conductivity and/or improved electrochemical properties (e.g., [7,8,9]). It is, therefore, natural that such materials have been considered as potential cathodes or solid electrolytes in prospective electrochemical devices for energy conversion and storage. Besides an application-oriented interest in such glass-ceramic nanomaterials, there is also a stress on the purely scientific aspect. Here, the focus is on determining and possibly also explaining the correlations observed between the microstructure of these nanomaterials and their electrical/electrochemical properties. Another important nanocrystallization-related issue is the observation that it is possible to stabilize high-temperature crystalline phases down to much lower temperatures if those phases form small crystalline grains embedded inside the original glassy matrix.

This paper presents a review of recent progress made in studies on the effects of thermal nanocrystallization of selected oxide glasses on their electrical properties. Another important point in these studies involves investigating and explaining the correlations between microstructure and electrical conductivity, especially in situations where nanocrystallization leads to a giant conductivity enhancement. A third important issue of interest is the phenomenon of the extension of the temperature range, even down to room temperature, in which some high-temperature crystalline phases can be present in the material. It should be underlined that in such a situation, the actual phase (out of several possible polymorphs) present may be strongly dependent on several factors, including the thermal history of the material. An application-oriented aspect of the studies is the synthesis of new glass-ceramic solid electrolytes or electrodes for energy conversion and storage devices like Li-, Na-ion all-solid batteries, or solid oxide fuel cells.

Nanostructured glass-ceramic materials described in this overview have been prepared by a two-stage process, involving: (i) synthesis of glasses of desired composition by standard melt-quenching and (ii) subjecting these glasses to thermal treatment at temperatures between the glass transition temperature *T_g_* and the crystallization temperature *T_c_*. Both temperatures have been determined by DTA or DSC analyses of the initial glasses. Usually, the temperature, duration and other syntheses’ parameters have been optimized to obtain the glass-ceramic samples with the highest conductivity, good microstructure (a large concentration of small nanometer-sized crystalline grains). These parameters were also optimized to extend the temperature range of the highly conducting high-temperature phases present in the material. The properties of as-synthesized materials in most publications have been studied by the following methods: electric conductivity (ionic, electronic, or mixed electronic-ionic) by impedance spectroscopy (IS), structure by X-ray diffractometry (XRD), thermal properties by differential thermal analysis (DTA) or differential scanning calorimetry (DSC), microstructure by scanning electron microscopy (SEM), transmission electron microscopy (TEM), and scanning transmission electron microscopy (STEM). In several cases, some additional techniques were used, e.g., nuclear magnetic resonance (NMR), to monitor the short-range order in glass-ceramics. Several glass-ceramic materials were tested as cathodes in laboratory-made electrochemical rechargeable cells. Experiments were conducted over a long period of time and using various experimental setups. More detailed information about the devices used in each experiment is given in the references provided in particular paragraphs.

## 2. Enhancement of Electrical Conductivity of Glasses by their Nanocrystallization

### 2.1. Enhancement of Ionic Conductivity

It was recognized a long time ago that heterogeneous systems containing an ionically conducting phase and a second phase, even an insulating one, can exhibit an enhanced overall conductivity. A classic example of such a case is the system LiI–Al_2_O_3_, in which an increased fraction of an insulating phase (Al_2_O_3_) dispersed in a modestly ionically conducting phase (LiI) led to a substantial increase of the electrical conductivity [7,8]. A similar effect was observed, e.g., in composites of Li_2_O–B_2_O_3_ [9]. In most of the similar cases, the conductivity increase in these systems was attributed to interfacial regions where the materials were highly disordered and exhibited enhanced ionic conductivity. The problem was extensively studied, experimentally and theoretically, by several groups. Overviews of those works can be found, e.g., in [8,10,11,12]. From those studies, it was clear that to enhance the conductivity in heterogeneous systems it is preferable to have a ramified network of interconnected thin interfacial regions. Such an opportunity is offered by the glass-ceramic processing in which one can control, by temperature and time of annealing, the crystallization process of initial glasses to obtain final heterogeneous materials with small crystalline grains embedded in glassy matrices.

One of the first papers reporting a substantial enhancement of the electrical conductivity of superionic conducting glasses after their thermal nanocrystallization was published by Adams et al. [13]. They showed, using a set of complementary experimental techniques, i.e., impedance spectroscopy (IS), X-ray diffractometry (XRD), and differential temperature analysis (DTA), that appropriate annealing of AgI-rich glasses of the AgI–Ag_2_O–V_2_O_5_ system at temperatures slightly higher than the glass transition temperature led to their partial crystallization (with emerging small crystalline grains of Ag_8_I_4_V_2_O_7_–ranging from ca 10 nm to over 100 nm, depending on the temperature of the heat treatment). This effect was correlated with a discernible enhancement (nearly four-fold) of their ionic (via Ag^+^ ions) conductivity. The authors also showed that additional annealing at higher temperatures led to a simultaneous exaggerated growth of the crystalline grains and an electric conductivity decrease below its initial value. As it was shown in a subsequent work of the same group [14], the maximum conductivity enhancement was observed when the estimated ratio surface area/volume of the crystallites attained its maximum. These observations indicated that the enhancement of the ionic conductivity of nanocrystallized glasses is due to the interfacial regions between the crystallites’ surfaces and the embedded glassy phase. 

Similar studies were carried out by Takahashi et al. [15] for another glassy system, namely, Li_2_O–V_2_O_5_–P_2_O_5_, at low V_2_O_5_ contents. The authors showed that appropriate heat treatment of the initial glasses led to their crystallization, accompanied by increased electrical conductivity. If the annealing temperature was too high, then the electrical conductivity decreased. The effects of heat treatment on the conductivity were complicated by the fact that the presence of V_2_O_5_ in the system could introduce some electronic component to the conductivity. Consequently, the materials exhibited mixed electronic conduction, with both components behaving differently on nanocrystallization: the ionic component decreased and the electronic one increased upon crystallization. One of the reasons why the crystallization did not cause an enhancement of the ionic conductivity is an exaggerated growth of crystalline grains. The latter phenomenon led to a decrease in the surface-to-volume ratio and consequently to lower fraction of interfacial regions in the final heterogeneous materials. As a result, the effective conductivity decreased. Other publications for the same Li_2_O–V_2_O_5_–P_2_O_5_ system, but for different compositions, reported a conductivity enhancement upon nanocrystallization due to the electronic component only (e.g., [16,17]). Studies on improved ionic conductors prepared by nanocrystallization of glasses were also undertaken by other research groups, e.g., by: Foltyn et al. [18] (for AgI-Ag_2_O-B_2_O_3_ system conducting via Ag^+^ ions), Nowiński et al. [19] (for AgI–Ag_2_O–P_2_O_5_ system conducting via Ag^+^ ions), Minami et al. [20] (for Li_2_S-based oxysulfide glasses conducting via Li^+^ ions), or Tatsumisago et al. [21] (Na^+^ ion conduction in sodium phosphosulfide Na_3_PS_4_ glass-ceramics). Some insight into the local and long-range structure of NASICON-like sodium glass-ceramics using a combination of NMR spectroscopy and XRD is presented in a recent paper by Eckert et al. [22]. Unfortunately, no information about the electrical conductivity is given in that paper. It should be added that NASICON is an acronym for natrium superionic conductor. The term was coined in the 1970s for a class of highly ionic conducting (via Na^+^ ions) solids (usually ceramics) of the composition A_1_B_2_(PO_4_)_3_, where A is a monovalent cation (eg. A=Na) and B is either a single or a combination of tri-, tetra-, and pentavalent ions (e.g., B = Zr, Si, Fe, ...). One of the pioneers of research on NASICON as a superionic conductor is Prof. J.B. Goodenough, the Nobel Laureate in Chemistry in 2019 [23]. These materials usually have open framework structures with mostly rhombohedral, but also monoclinic unit cells. Since the late 1970s, they have been tested together with beta-aluminas as solid electrolytes in Na–S rechargeable batteries. Today, many electronic or mixed electronic ionic conductors, suitable for cathodes in rechargeable batteries (e.g., Li-ion or Na-ion), have the same structure [24]. Another example of the ionic conductivity enhancement resulting from glass-ceramic processing was presented by Honma et al. [25]. They showed that the annealing of composites of NASICON with glasses of the nominal composition xNa_2_O–(70−x)Nb_2_O_5_–30P_2_O_5_ at 900 °C for 10 min led to their partial crystallization with crystallites of a NASICON-like structure interpenetrated by the residual glassy phase. The conductivity of these final glass-ceramic composites was higher than that of the initial material by a factor of 40 [25]. The increase was attributed to both dense NASICON ceramics and the interfacial regions.

In most of the above studies, it was concluded that the enhanced conductivity of glass-ceramic materials was due to better conducting interfacial regions at newly formed crystalline grains embedded in the glassy matrices. In some cases, the observed conductivity increase was attributed to the formation of highly conducting crystalline grains (e.g., [26]). It was also found out that the excessive growth of crystalline grains in these materials led to a decrease in the ionic conductivity because of a lower volume share of the better conducting interfacial regions. All these phenomena are consistent with a model of space-charge interfacial regions proposed and analyzed by Maier [8]. Heitjans and Indris [9] proposed an alternative theoretical model explaining the role of interfaces in the overall conductivity in nanocomposite systems. In this “atomistic” model they considered the atomic slabs near the interfaces and wanted to learn from numerical simulations what the main factor of the conductivity enhancement is—a thermodynamic one (higher concentrations of defects at the interfaces) or a kinetic (lower energy barriers for local ionic hopping). Confronting the results of DFT (density functional theory) modeling with experimental data on Li_2_O:B_2_O_3_ composites they concluded that an increased concentration of defects near the interfaces (i.e., the thermodynamic factor) plays a dominant role in those systems. 

The research on the nanocrystallization of ionic conducting glasses has shown that these materials are promising alternatives to crystalline solid electrolytes for all-solid Li-, Na-, and Ag-ion batteries [27]. 

### 2.2. Enhancement of the Electronic Conductivity

Nanocrystallized ionically conducting glasses, described in the previous section, can be prospective solid electrolytes for all-solid batteries [27,28]. Similarly, glass-ceramics with enhanced predominantly electronic conductivity and good electrochemical properties can be attractive as electrode materials for a wide range of electrochemical devices for energy conversion and storage, including Li- or Na-ion all-solid rechargeable batteries. The advantages of glass-ceramic materials for energy-related applications over other materials of the same composition, but prepared by other methods, include the low cost of materials and processing, the possibility of preparing dense, non-porous and highly conducting materials. Moreover, there is no need to use any conductivity-improving foreign additives (such as, e.g., graphite). The latter factor is an obvious advantage of the nanocrystallization method over the standard synthesis of cathode materials for Li- or Na-ion batteries, which require an addition of carbon to improve the electronic conductivity and generally results in low-density materials. When optimized, the glass-ceramic method can be an important alternative to other methods of synthesizing electrode (mostly cathode) materials for the large market of rechargeable batteries. 

There are several systems, interesting as potential cathodes in rechargeable batteries and other electrochemical devices, which can be produced by glass-ceramic processing. One of the most interesting and extensively studied of them is the V_2_O_5_–P_2_O_5_ system. Vanadates and vanadate-rich systems are very interesting for many applications, but also for purely scientific reasons (e.g., [29,30]). 

#### 2.2.1. Electronic Conductivity Enhancement in V_2_O_5_–P_2_O_5_ System

In this system, V_2_O_5_ is responsible for the electronic conductivity (via V^4+^/V^5+^ hopping centers) and electrochemical activity (intercalation/deintercalation of lithium). P_2_O_5_ acts as a supporting glass former which facilitates the preparation of glasses by a standard melt-quenching technique. The studies on the electrochemical properties of polycrystalline vanadate-based cathodes in Li-ion batteries, prepared by standard solid-state reactions were summarized e.g., in references [31,32]. There are also a number of studies on thermal nanocrystallization of vanadate-phosphate glasses and their consequences for structure, microstructure, thermal properties, and electrical conductivity of the resulting glass-ceramics (e.g., [33,34,35]). In a series of these studies, the chemical composition of these glasses was set to 90V_2_O_5_⋅10P_2_O_5_. The high content of V_2_O_5_ ensured that the electronic properties of these materials were entirely due to the vanadate component (pure phosphate materials are electronic insulators). 

**Figure 1:**
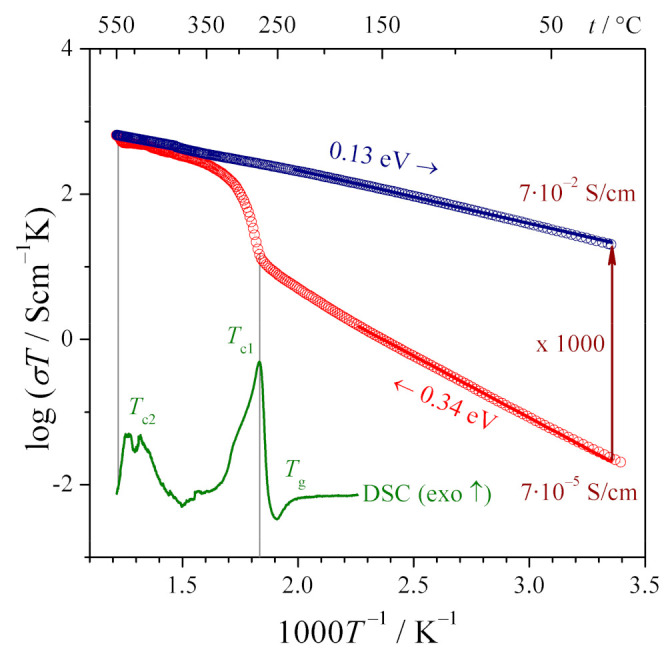
Temperature dependence of the electronic conductivity of a 90V_2_O_5_⋅10P_2_O_5_ glassy material upon heating to 550 °C (1 °C/min.) and cooling down to RT. The significant and irreversible increase in the conductivity is a result of nanocrystallization above T_c_. The inset shows a corresponding DSC trace (Reproduced with permission from ref. [34]. Copyright 2014, Elsevier).

The as-synthesized 90V_2_O_5_⋅10P_2_O_5_ glasses, before the measurements of their electrical conductivity, firstly had been studied by thermal analyses [33]. The goals of the thermal analyses were two-fold: (i) an independent confirmation of the amorphous state of the as-synthesized glasses (in conjunction with XRD experiments), and (ii) determination of the temperatures of the glass transition *T_g_* and crystallization *T_c_*. The information on *T_g_* and *T_c_* was necessary to determine the temperature range of the heat treatment, in which the glasses undergo an optimal nanocrystallization (possibly a high concentration of nanosized crystalline grains embedded in the glassy matrix), and the conductivity reaches its maximum enhancement. A series of nanocrystallization processes confirmed that an optimum heat treatment leads to a substantial irreversible conductivity enhancement, as shown in Figure 1. The conductivity of the original glassy material is low (7 × 10^−5^ Scm^−1^ at room temperature). In the heating stage (red circles in Figure 1), it initially increases according to the Arrhenius formula with the activation energy of 0.34 eV until ca 270 °C, where it undergoes a rapid non-Arrhenian enhancement. At higher temperatures, it continues to increase according to the Arrhenius formula but with the activation energy much lower than 0.34 eV. With the cooling ramp, the conductivity decreases following the Arrhenius formula with the activation energy of 0.13 eV. One could also observe a substantial increase (by a factor of 1000) in the conductivity at room temperature. These conductivity changes are closely correlated with the crystallization processes, indicated by the thermal events visible on the DTA trace, shown as the inset in Figure 1. It was shown that the temperature dependence of the conductivity of the sample in the 2nd heating run is the same as during the 1st cooling stage. This means that the electrical properties of the thermally treated, partly crystallized samples remain stable in the temperature range 20–550 °C. 

**Figure 2:**
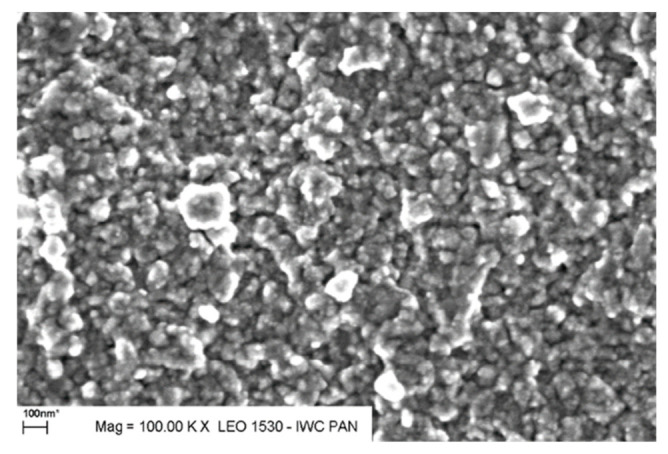
SEM micrograph of a 90 V_2_O_5_∙10P_2_O_5_ glassy sample after nanocrystallization at 275 °C (Reproduced with permission from ref. [35]. Copyright 2011, Elsevier).

This irreversible conductivity enhancement was also closely correlated with the change of the microstructure. A SEM micrograph taken at 275 °C (Figure 2) shows that the nanocrystallized 90V_2_O_5_⋅10P_2_O_5_ glass-ceramics consists of evenly distributed small crystallites (below 100 nm in size, identified by XRD as α–V_2_O_5_—orthorhombic, space group Pmmn [36]) embedded in the glassy matrix. A large concentration of small crystalline grains is advantageous from the viewpoint of the role of interfaces in the effective conductivity of the material because small grains lead to high surface-to-volume ratios.

**Figure 3:**
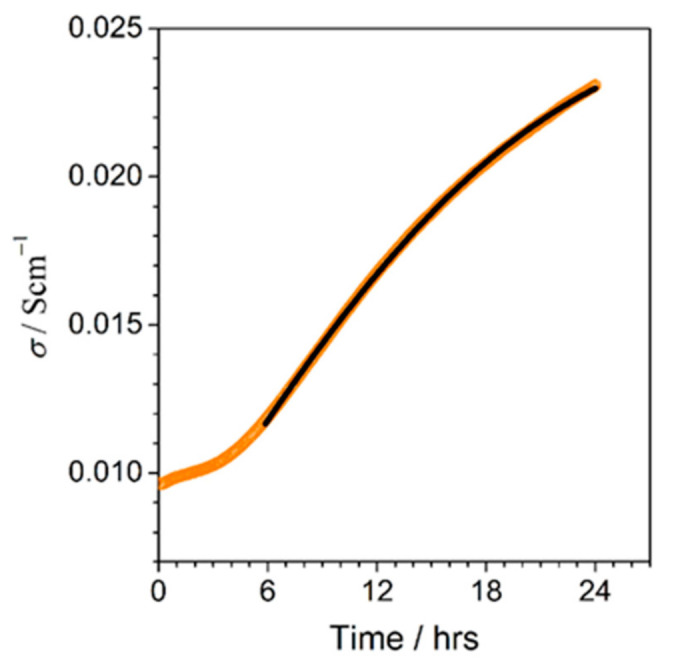
Conductivity increase vs. time during the isothermal annealing of a 90 V_2_O_5_·10P_2_O_5_ glassy sample at 240 °C (orange circles) with a fit (solid black line) (Reproduced with permission from ref. [37]. Copyright 2013, Elsevier).

Another way to observe a substantial increment in electronic conductivity consisted of isothermal annealing of the materials at temperatures close to *T_g_*. The observed time dependence of the conductivity of the 90V_2_O_5_⋅10P_2_O_5_ glass during such an experiment carried out at 240 °C shows its monotone increase, which is in line with the progress of the formation of crystallites inside the glassy matrix (Figure 3). 

**Figure 4:**
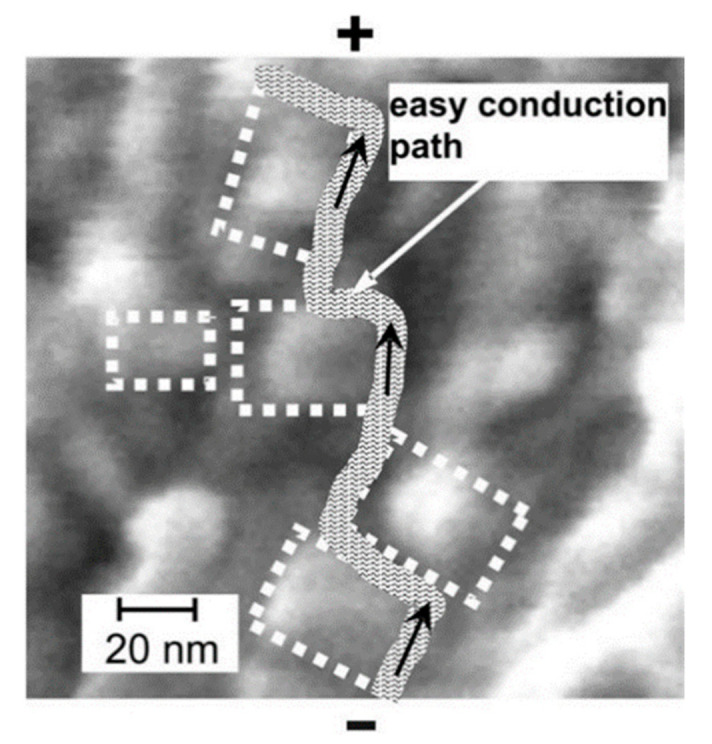
SEM image of a nanocrystallized 90 V_2_O_5_·10P_2_O_5_ glass with a sketch of an easy conduction path along the interfacial regions next to the crystallites (lighter spots) (Reproduced with permission from ref. [35]. Copyright 2011, Elsevier).

The experimental time dependence of the conductivity shown in (Figure 3) was successfully fitted by a formula derived by the authors from a core-shell model and the Avrami approach (e.g., [38]). The formula reads [37]:

(1) $$\sigma \left(t\right)=\sigma_{g}+\left(\sigma_{n}-\sigma_{g}\right)\cdot \left[1-\exp{\left(-K{\left(t-t_{0}\right)}^{n}\right)}^{2/3}\right],$$

where $\sigma_{g}$, $\sigma_{n}$ represent the electronic conductivities of the glassy and the nanocrystallized sample (in S/cm), respectively, *n* is the exponent, related to the dimensionality of the process [39], *K* is a constant depending on the crystallization rate (in hr^−n^), and *t*_0_ is the time (expressed in hours) when crystallized phase starts to be detectable. The exponent of 2/3 in the above formula is the ratio of the dimensionalities of the shell (2D) and the bulk (3D). The core-shell model assumes that each crystallite (core) is surrounded by a thin shell, whose structure and properties are different from those of both crystallites and the glass matrix. The defective nature of this shell leads to the formation of ionic defects (e.g., oxygen vacancies), which is accompanied by the appearance of small polarons (e.g., [40]). In the case of a large concentration of small crystallites, the individual shells around crystallites overlap and consequently they form easy conduction paths for the small polarons). The idea of the model of the electronic transport inside such a glass-ceramic material is shown in Figure 4. Easy conduction paths circumvent individual crystalline grains (lighter spots), as shown on a magnified SEM micrograph of the 90V_2_O_5_⋅10P_2_O_5_ glass-ceramics. 

It is well established that the electronic conduction in vanadate-based glasses occurs via a small-polaron hopping mechanism. According to Mott’s theory [41], the electric charge transport at high temperatures ($T>\theta_{D}/2)$, where $\theta_{D}$ denotes Debye temperature), is assisted by phonons. 

The corresponding expression for conductivity in this temperature range is given by the formula (2) [41]:

(2) $$\sigma \left(T\right)=\nu_{el}\cdot c\left(1-c\right)\cdot \left(\frac{e^{2}}{R\cdot k_{B}T}\right)\cdot \exp\left(-2\alpha R\right)\cdot \exp\left(-\frac{E_{a}}{k_{B}T}\right)$$

where $\nu_{el}$ denotes electron attempt frequency, *c* is the fraction of a given valence state of a transition metal (e.g., in the case of vanadium $c=\left[V^{4+}\right]/\left(\left[V^{4+}\right]+\left[V^{5+}\right]\right)$, *e* denotes elementary charge, α wave function decay distance, *R* the average distance between hopping centers, *E*_a_ the activation energy of electronic (polaronic) conduction, *T* temperature, *k_B_* Boltzmann constant. The theoretical expression for the activation energy includes a component $W=W_{0}\left(1-r_{p}/R\right)$, where *W*_0_ is a constant and *r_p_* denotes a radius of small polaron [41].

**Figure 5:**
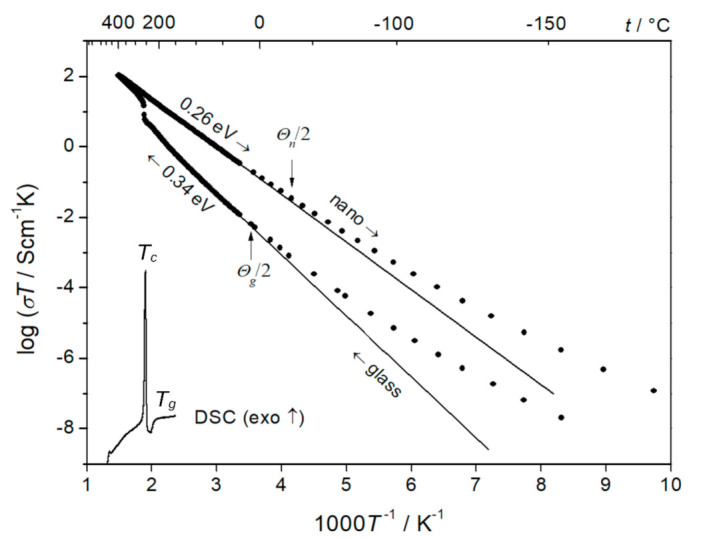
Temperature dependences of conductivity of 90V_2_O_5_·10P_2_O_5_ glass and nanomaterial in an extended temperature range from −170 °C up to +400 °C. A DSC run taken on heating is shown for comparison. (Reproduced with permission from ref. [35]. Copyright 2011, Elsevier).

At low temperatures $T<\theta_{D}/4$, when phonons are “frozen”, the transport mechanism changes to the “variable range hopping”, which at very low temperatures follows the famous “*T*^–1/4^” formula. The experimental temperature dependencies of the conductivity of both 90V_2_O_5_·10P_2_O_5_ initial and final glass-ceramics deviate at low temperatures from the Arrhenius behavior (Figure 5) and agree with the famous Mott’s “T^–1/4^” law: $\sigma \left(T\right)=A\exp\left(-B/T^{1/4}\right)$ [41]. It was shown that the conductivity in the low-temperature range follows that law. The fact that the low-temperature conductivity of both the glassy phase and the final glass-ceramic material follows the “T^–1/4^” law proves that in both cases (i.e., in glass and in glass-ceramics) in this temperature range, the conductivity mechanism is the same–the variable range hopping of small polarons. Additionally, the fact that the conductivity of the final glass-ceramics is higher than that of the initial glass, is consistent with the hypothesis that the easy conduction paths in the interfacial regions contain a higher concentration of aliovalent pairs of V^4+^-V^5+^ hopping centers, compared to both the glassy phase and the crystallites. A decrease in the distance between such centers leads to an increase in conductivity and a decrease in activation energy. This observation agrees with the Mott formula, which predicts a conductivity enhancement for a shorter average distance between the hopping centers. 

It should be noted that small polarons, responsible for the electronic transport in many solids, usually appear in conjunction with some defects. For example, in the case of CeO_2_ small polarons appear together with oxygen vacancies. When an oxygen vacancy is formed in CeO_2_, it leaves two electrons [40]. These electrons prefer to localize on Ce atoms neighboring the vacancy, as small polarons (Ce^3+^). Therefore the concentration of these small polarons is correlated with the concentration of oxygen vacancies, which may depend on local oxygen levels [40]. In the case of 90V_2_O_5_·10P_2_O_5_ glasses and glass-ceramics all synthesis steps and all measurements were carried in the air [35]. Moreover, both glassy and glass-ceramic samples were dense, non-porous and therefore, their interiors, including interfacial regions, were not exposed to the ambient air. No effects suggesting any discernible coupling between polaronic transport and potential ionic conduction via oxygen vacancies have been detected.

#### 2.2.2. Electronic Conductivity Enhancement in Li–V–Fe–P–O and Other Systems

Another group of electronically conducting glass-ceramics for potential use as cathodes for Li-ion batteries are analogs of crystalline lithium phospho-olivine of the composition LiFePO_4_. This crystalline material was discovered by J.B. Goodenough et al. [42] as an inexpensive, safe and efficient alternative of commonly used unsafe, toxic and expensive LiCoO_2_ cathode material in commercial Li-ion batteries. LiFePO_4_ has excellent intercalation properties, but its poor electronic conductivity (ca 10^−9^ Scm^−1^ at room temperature [43]) has been much too low for mass-scale applications. Therefore, many synthetic routes have been proposed to overcome this problem. Usually, the research groups have carried out a variety of syntheses of LiFePO_4_ with an addition of thin layers of highly conducting carbons to improve the conductivity. Glass-ceramic processing, overviewed in this paper, is a different route to prepare highly conducting LiFePO_4_-based dense materials without any additives. Its basic two-step strategy consists firstly in preparation of glassy samples of the system Li_2_O–FeO–V_2_O_5_–P_2_O_5_ with compositions close to that of LiFePO_4_. In the next step, the as-prepared glasses undergo a thermal treatment to obtain the glass-ceramic microstructure consisting of a large concentration of nanosized crystalline grains of the desirable electrochemically active LiFePO_4_ phase, the residual glassy matrix and the interfacial regions (presumably highly conducting) [44,45]. The same strategy has been used for vanadate-phosphate glasses, as described above. In the case of amorphous analogs of LiFePO_4_ a small addition of V_2_O_5_ was necessary to improve glass-forming properties of the starting mixtures. Fortunately, this additive is electrochemically active and could enhance the electrochemical properties of the final material. It is interesting to note that the crystallization of multicomponent glasses containing FeO–P_2_O_5_ often leads to the appearance of crystallites of LiFePO_4_. This observation was addressed by Hirose et al. [46]. They studied glasses of the composition 26Li_2_O·43FeO·5Nb_2_O_5_·26P_2_O_5_ and glass-ceramic materials prepared by appropriate annealing containing a high fraction of LiFePO_4_. In conclusion of their studies, including, e.g., Mössbauer spectroscopy, the authors suggested that the main reason for the formation of LiFePO_4_ rather than other crystalline phases during annealing was the similarity of valence states and local environment of iron in the original glasses and in LiFePO_4_ crystals [46].

**Figure 6:**
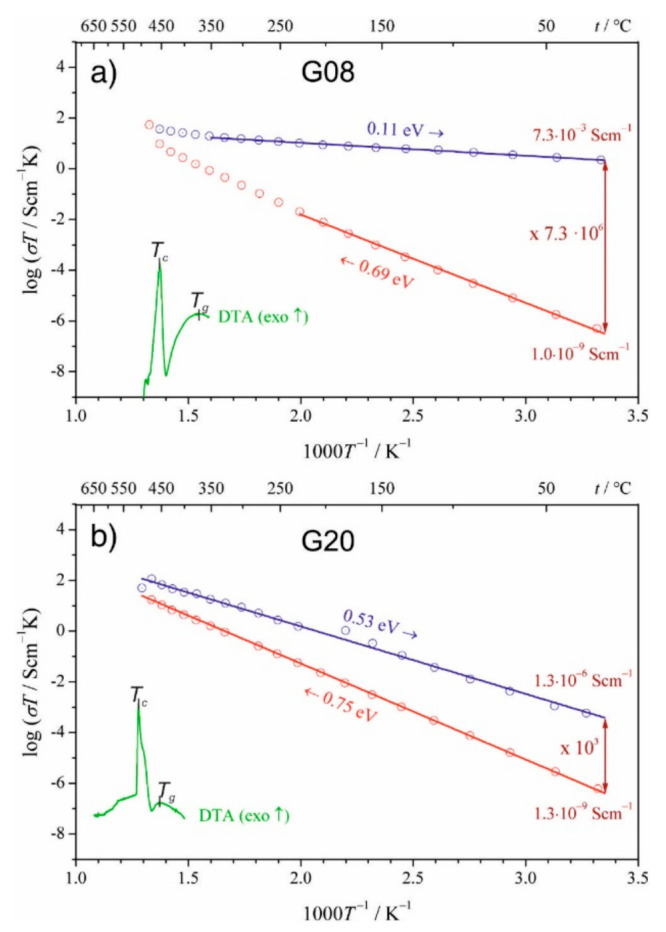
Arrhenius plots of the electrical conductivity for olivine-like materials: (**a**) LiFe_0.80_V_0.08_PO_4_ (G08) and (**b**) LiFe_0.50_V_0.20_PO_4_ (G20), before (heating ramps–red) and after nanocrystallization (cooling ramps–blue). The respective DTA traces recorded at a rate of 1 °C/min are inserted for comparison. (Reproduced with permission from ref. [45]. Copyright 2015, Elsevier).

The results presented above were obtained for samples in which mole fractions of vanadium were 0.08, 0.10, 0.15 and 0.20. Those samples were labeled: G08, G10, G15, and G20, respectively. As can be seen in Figure 6a, even small changes in the vanadium content have an important impact on the electronic conductivity of those materials. 

In the case of the G08 material, with low vanadium amount, all analyzed crystalline grains (less than 20 nm) were identified as LiFePO_4_. In materials with higher vanadium contents (i.e., G15 or G20) the dominating crystalline phase was still LiFePO_4_ olivine, but one could detect and identify grains of the NASICON-like Li_3_V_2_(PO_4_)_3_ phase [47]. Moreover, it was empirically evidenced that, for higher fractions of vanadium (compositions G15 and G20), the heat treatment leads to an excessive growth of crystallites (up to ca 100 nm) accompanied by a substantial decrease in the electronic conductivity of the materials. The latter phenomenon is caused by a drastic reduction of the ratio of the surface area of the crystallites to their volume due to the increase in their size. 

The idea of nanocrystallization of the glassy analogs of LiFePO_4_ has proven to be effective. The main goal: a substantial conductivity enhancement, was achieved. The temperature dependence of the conductivity, in this case, is presented in Figure 6a (for the material with lower vanadium content G08). One should notice an extremely high enhancement (by a factor 7.3 × 10^6^) of the conductivity at room temperature, following the glass-ceramic processing and a substantial decrease in the activation energy from initial 0.69 eV for the glass to 0.11 eV for the final glass-ceramic material (Figure 6a).

**Figure 7:**
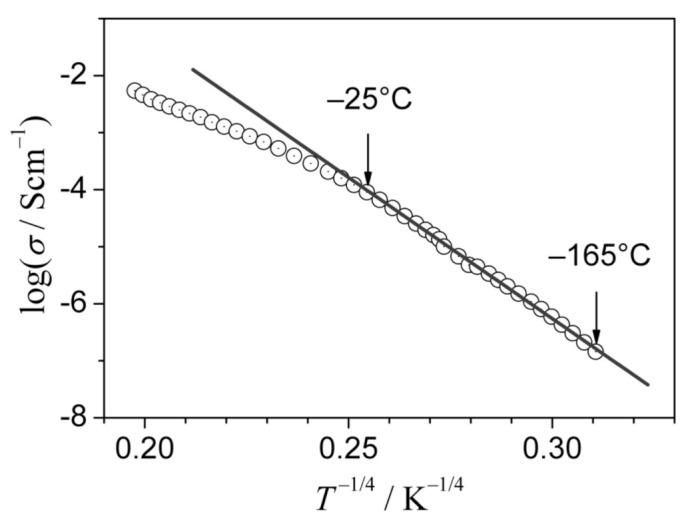
Conductivity of the nanocrystallized LiFe_0.75_V_0.1_PO_4_ material (G10) at low temperatures in Mott’s coordinates $\log\sigma \;\mathrm{vs}.\;T^{-1/4}$. The solid line is a linear fit to the experimental data (circles) (Reproduced with permission from ref. [44]. Copyright 2013, Elsevier).

To identify the mechanism of the electronic transport in the nanocrystallized glass-ceramic samples under study, the conductivity measurements were extended down to ca. −165 °C. The temperature dependencies of the conductivity in the low-temperature range visibly deviated upwards from the Arrhenius behavior, similarly to the case of 90V_2_O_5_ ∙10P_2_O_5_ materials (cf. Figure 5). This feature confirms that the electronic conduction in this material at low temperatures occurs via a small polaron hopping. As can be seen in Figure 7, the temperature dependence of conductivity follows the “$T^{-1/4}$” law predicted by the Mott theory for small polaron hopping in glassy materials [41]. From the slope of the dependence shown in Figure 7, it was possible to determine the density of states at the Fermi energy *N*(*E_F_*) = 1.6 × 10^20^ eVcm^−3^, which is important for the theoretical description of the small polaron hopping. 

**Figure 8:**
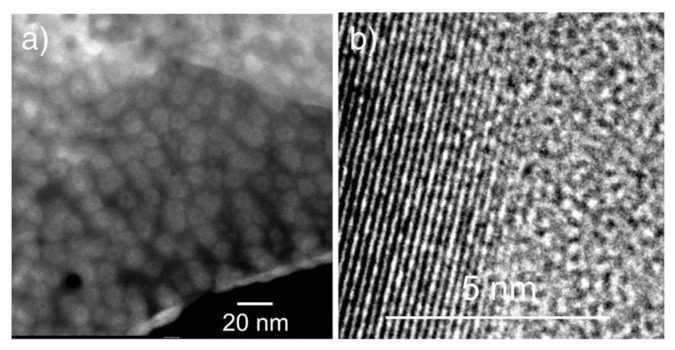
Microstructure of different fragments of glassy sample LiFe_0.80_V_0.08_PO_4_ (denoted as G08) nanocrystallized at 480 °C: (**a**) STEM image of nanograins closely packed in a glassy matrix and (**b**) a magnified HR-TEM image of an interface between a nanograin and a glassy phase [Reproduced with permission from ref. [45]. Copyright 2015, Elsevier.].

**Figure 9:**
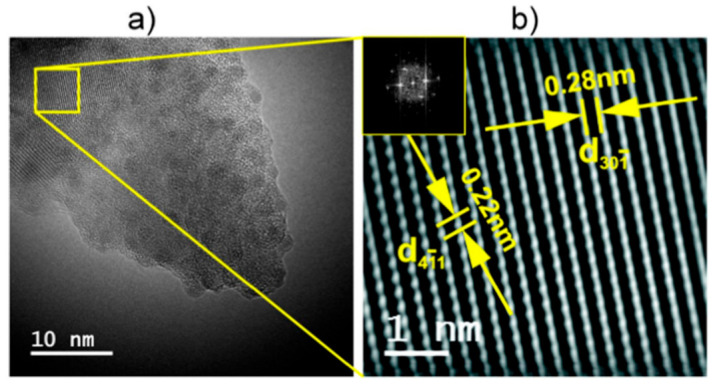
HR-TEM images of a sample LiFe_0.80_V_0.08_PO_4_ (G08) heated up to 493 °C: (**a**) a HRTEM image with a crystalline area selected for analysis (a box ), (**b**) a Fourier filtered image of the area marked in (**a**) with inter-plane distances of the LiFePO_4_ (Reproduced with permission from ref. [47]. Copyright 2017, Elsevier).

**Figure 10:**
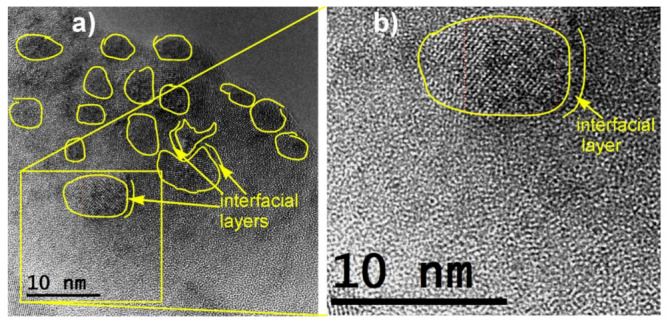
HR-TEM micrograph of a sample LiFe_0.5_V_0.20_PO_4_ (G20) heat-treated up to 463 °C (in argon): (**a**) marked are several “grains” with crystalline structure–most of the material remains amorphous; (**b**) a magnified fragment of the image (**a**) with a discernible ordered grains and a fragment of an intermediate interfacial layer around the grain (Reproduced with permission from ref. [48]. Copyright 2015, Elsevier).

**Figure 11:**
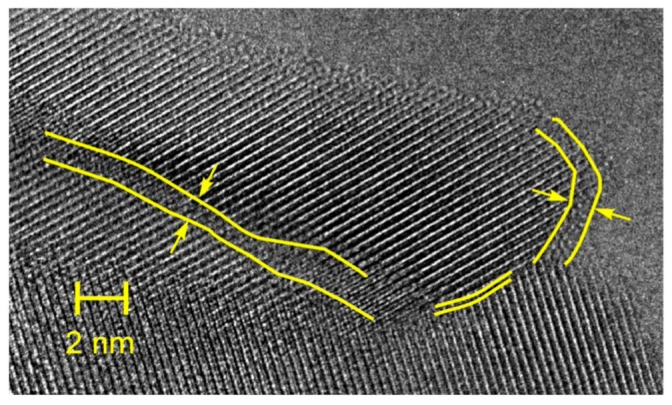
Magnification of a fragment of the HR-TEM image of a LiFe_0.5_V_0.20_PO_4_ (denoted as G20) specimen heated up to 493 °C. Visible are nanosize grains and interfacial regions around them (some of them marked) (Reproduced with permission from ref. [47]. Copyright 2017, Elsevier).

Similarly to the case of vanadate-phosphate glass-ceramics, also in the case of nanocrystallized glasses of the Li_2_O–FeO–V_2_O_5–_P_2_O_5_ system, their substantial conductivity enhancement, caused by thermal treatment, is strongly correlated with their dual microstructure. HR-TEM and STEM images (Figure 8, Figure 9, Figure 10 and Figure 11) clearly show that the samples under study are glass-ceramic composites, containing crystalline grains of LiFePO_4_ (and a smaller fraction of Li_3_V_2_(PO_4_)_3_), typically 5–100 nm in size, embedded inside the residual glassy matrix. In this situation, the large concentration of small crystalline grains (average size of ca 5 nm; Figure 8a) causes the interfacial “conduction tissue” to be ramified and can greatly contribute to the overall conductivity. It should be underlined that the conductivity of crystalline phase inside grains (LiFePO_4_) is low, typically 10^−7^ Scm^−1^ at room temperature [43]. The conductivity of the glassy matrix is even lower [45].

EDX analyses showed that vanadium acts as a dopant inside a glassy phase for compositions with low vanadium contents (i.e., LiFe_0.80_V_0.08_PO_4_). XRD patterns showed that the only crystalline phase detected is LiFePO_4_. The highest conductivity was observed for the material with low vanadium content, i.e., LiFe_0.80_V_0.08_PO_4_ (G08), in which the crystalline grains of LiFePO_4_ were small (ca 10 nm in average). The interfaces between amorphous and nanocrystalline regions for samples with different vanadium contents are visible in Figure 8a, Figure 10 and Figure 11. The defective and strongly disordered chemically and structurally interfacial regions are responsible for the giant increase in the electronic conductivity of the final glass-ceramic materials. In an HR-TEM micrograph of the thermally treated material with low vanadium content (G08) (Figure 9) one was able to determine the interplanar distances, characteristic for the phospho-olivine structure of LiFePO_4_ [47]. Our independent XRD studies of the same material confirmed the presence of the same crystalline phase.

**Figure 12:**
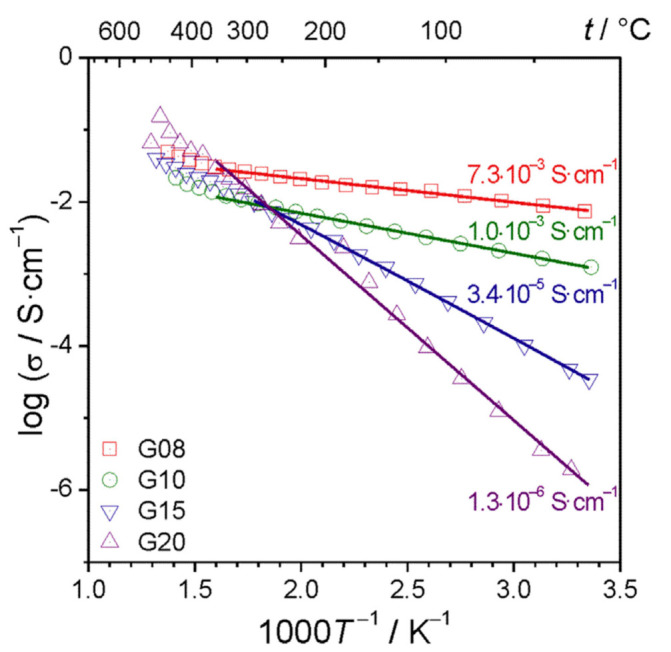
Temperature dependences of the electrical conductivity of the nanocrystallized glasses of the Li_2_O–FeO–V_2_O_5_–P_2_O_5_ system: G08 (LiFe_0.80_V_0.08_PO_4_), G10 (LiFe_0.75_V_0.10_PO_4_), G15 (LiFe_0.625_V_0.15_PO_4_) and G20 (LiFe_0.50_V_0.20_PO_4_). (Reproduced with permission from ref. [48]. Copyright 2016, Elsevier).

The microstructure of the nanocrystallized sample with higher vanadium content (G20) shown in Figure 10 contains a lower concentration of crystalline grains and a higher fraction of the amorphous phase. This type of morphology combined with larger average grain sizes is apparently responsible for the low conductivity of those materials, the lowest among all the compositions under study (Figure 12). 

**Figure 13:**
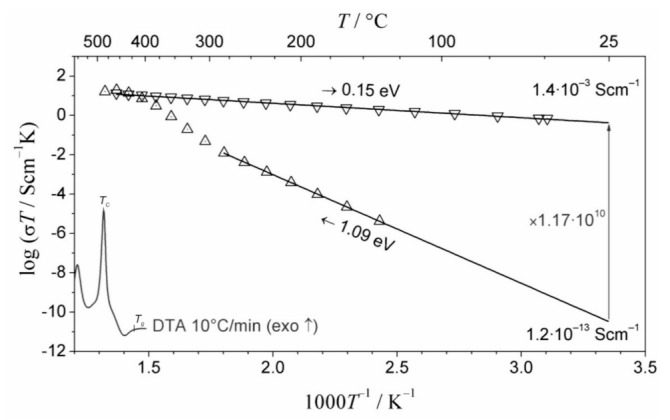
Dependence of electrical conductivity of Li(Fe_1–x_Mn_x_)_0.88_V_0.08_PO_4_ (x = 0.5) sample upon heating to 480 °C and subsequent cooling to room temperature. DTA trace of the material is shown in the inset. (Reproduced with permission from ref. [49]. Copyright 2017, The Electrochemical Society).

It is noteworthy that the conductivity at the end of the process (10^−3^ Scm^−1^ at room temperature) is acceptable for cathode operation in a Li-ion cell. Such a high conductivity enhancement could raise doubts about its origin. Among possible causes, one can be replaced: the formation of a metallic phase (e.g., iron phosphide) inside the glass-ceramic material, an effect of so-called metal-insulator transition (MIT), or possible carbon impurities. None of these hypothetical causes of the conductivity enhancement was proved to be effective in this case [45]. In particular: i) no traces of iron phosphide were detected in the XRD patterns, ii) the conductivity increased with temperature, which is opposite to the metallic behavior, iii) the MIT effect is reversible, whereas the conductivity changes, in this case, were irreversible, and finally, iv) no carbon was added in the glass-ceramic processing. Therefore, the only plausible origin of this phenomenon (a huge conductivity increase upon nanocrystallization) is an increase in the concentration of the V^4+^-V^5+^ and Fe^2+^-Fe^3+^ hopping centers in the interfacial regions the glass-ceramics [45]. The idea of multi-channel electron hopping was also used in olivine-like nanocrystallized glasses in which a part of iron had been substituted by manganese [49]. In that system, additional Mn^2+^-Mn^3+^ hopping centers appear, contributing to a considerable conductivity increase by a factor of 10 orders of magnitude (Figure 13).

Boron oxide (B_2_O_3_), along with P_2_O_5_ and SiO_2_, belongs to the best oxide glass formers. In several papers, studies to verify whether borate glasses can undergo thermal nanocrystallization were reported. The investigation started with the Li_2_O–FeO–B_2_O_3_ system [50], because one can prepare glassy analogs of LiFeBO_3_. It is noteworthy that this material resembles LiFePO_4_, an important cathode for commercial batteries. Crystalline LiFeBO_3_ is also recognized as a cathode material with a theoretical gravimetric capacity of 220 mAh/g. It also suffers from modest electrical conductivity, as low as ca. 10^–7^ S/cm at room temperature [51]. Different samples were heat-treated up to various temperatures between *T_g_* and *T_c_*, what led to an irreversible conductivity enhancement. The best results (σ_nano_ = 1.4·10^–5^ S/cm, *E_a_* = 0.18 eV) were obtained for heating up to 475 °C. Even though the final value is not spectacular, it still means an increase by 10^6^ in comparison to the pristine glass, and factor of 10^2^ compared to polycrystalline material. It was evidenced that conductivity values of glassy and nanocrystallized samples were not due to iron oxide precipitates. Grain sizes of FeBO_3_ and LiFeBO_3_, estimated using the Scherrer formula, were 45–50 nm and 70–80 nm, respectively. 

Impedance spectra collected in the full temperature range during heating and cooling ramps, regardless of temperature, consisted of two arcs. They were attributed to electronic and ionic components of the conductivity. The contribution from ionic conductivity was higher at elevated temperatures than at low temperature, because ion mobility (and overall ionic conductivity) increases significantly with growing temperature. Studies on other borate systems followed these studies, i.e., Li_2_O–MnO–B_2_O_3_ [52], Li_2_O–FeO–MnO–B_2_O_3_, and Li_2_O–FeO–V_2_O_5_–B_2_O_3_ [53]. The common issue with nanocrystallization of borate glasses is the effect of dopants on the properties of the final nanomaterials. The general observation was that samples with higher Mn content showed better glass-forming properties, whereas samples with higher Fe content exhibited higher conductivity of the final nanomaterials. 

Similar studies on the conductivity, structure, microstructure, thermal properties of glass-ceramics prepared by thermal treatment were carried out for several other systems: BaO–V_2_O_5_–Bi_2_O_3_ [54], Fe_2_O_3_–PbO_2_–Bi_2_O_3_ [55], Fe_2_O_3_–PbO_2_–TeO_2_ [56], and BaO–TiO_2_–V_2_O_5_–Bi_2_O_3_ [57]. In these systems, one could see a substantial enhancement of the electronic conductivity upon nanocrystallization of the initial glasses. In the case of BaO–V_2_O_5_–Bi_2_O_3_ system the conductivity increased by a factor of over 10^4^ [54], for Fe_2_O_3_–PbO_2_–TeO_2_ the increase was about 10^4^, and for Fe_2_O_3_–PbO_2_–Bi_2_O_3_ it was over 5 × 10^4^ [55]. For the BaO–TiO_2_–V_2_O_5_–Bi_2_O_3_ system the conductivity enhancement factor was between 10 for low contents (5 mol%) of BaTiO_3_ to over 6 × 10^4^ for 15 mol% of BaTiO_3_ [57]. In all cases this effect was attributed to an increased concentration of pairs of aliovalent ions of vanadium (V^4+^/V^5+^) or iron (Fe^2+^/Fe^3+^), in the interfacial regions of the respective glass-ceramic materials. These pairs serve as hopping centers in the small-polaron hopping mechanism of electrical conduction. A typical average distance between the hopping centers was estimated as 0.38 nm (in Fe_2_O_3_–PbO_2_–TeO_2_ materials) [56]. Another important parameter is the density of states (DOS) at the Fermi level *N*(*E_F_*). In the latter system its value was close to 10^20^ eVcm^−3^ [56], which is similar to *N*(*E_F_*) calculated in the cases of glass-ceramic materials in V_2_O_5_–P_2_O_5_ and Li_2_O–FeO–V_2_O_5_–P_2_O_5_ systems (ca 1.6 × 10^19^ eVcm^−3^ [35] and 1.6 × 10^20^ eVcm^−3^ [44], respectively). The increased concentration of hopping centers in the interfaces leads to formation of “easy conduction paths” in these regions, which can dominate the effective conductivity of the glass-ceramic material (e.g., [56]).

Some research was done on glass-ceramic processing and its effects on the electrical conductivity of materials of 22.5BaTiO_3_⋅7.5PbTiO_3_⋅70V_2_O_5_ composition [58]. Its results show that the annealing of the original glasses at optimum temperature of 723 K causes an increase in their electronic conductivity (due to the V_2_O_5_ component) by more than 3 orders of magnitude. The study showed that the conductivity increase was correlated with changes in the microstructure, where one could observe the appearance of small crystallites (mainly Ba_2_Ti_9_O_20_ and V_2_O_5_) inside the glassy matrix. The conductivity of the final material was higher by the factor of 10 than that of the original glass. The increase was attributed to enhanced hopping rates of small polarons within disordered and ramified interfacial regions [58]. In a very recent paper by Mogus-Milankovic et al. [59] it was shown that the appropriate annealing (935 °C, 12 h) of glasses of the binary system WO_3_–P_2_O_5_ led to a increase in conductivity at room temperature from 4.3 × 10^−6^ to 1.6 × 10^−4^ Scm^−1^. They attributed that increase mainly to formation of nanoscopic (ca 80 nm) crystallites of semiconducting W_2_O_3_(PO_4_)_2_ and WO_3_ [59].

Other studies on applying the glass-ceramic process to synthesize materials with improved electrical conductivity have been carried out on a binary 40Fe_2_O_3_·60P_2_O_5_ system [60]. The authors observed that an appropriate annealing program of initial glasses leads to formation of dual glass-ceramic microstructure. The crystalline phases were identified as Fe_4_(P_2_O_7_)_3_ and Fe(PO_3_)_3_. These morphological changes were accompanied by a 10-fold increase of the electrical conductivity. The authors ascribed that increase partly to the presence of electrically conducting Fe_4_(P_2_O_7_)_3_ phase but also to the interfacial regions. In both cases the enhanced electronic transport occurred by a small polaron hopping mechanism between aliovalent Fe^2+^-Fe^3+^ centers [60].

## 3. Extension of Thermal Stability Range of High-Temperature Phases by Controlled Nanocrystallization of Glasses

Many crystalline superionic conductors exhibit polymorphism and, depending on the external conditions (pressure, temperature), can adopt different crystalline structures, with different physical properties, including the electrical conductivity. In some of them a highly conducting phase is stable at high temperature only, which limits the practical application of these ionic conductors as solid electrolytes in electrochemical power sources and fuel cells. Therefore, it has been natural to search for synthetic procedures to extend the temperature stability range of the highly conducting high-temperature phases downwards, even to room temperature. In the following sections, there are presented two important examples of such ionic conductors and a nanocrystallization-based synthesis method, which has proven to be efficient in stabilizing high-temperature phases down to room temperature. In all these considerations, it is important to pay attention to the polymorphism of the compounds under study. The same compound may adopt different crystalline structures depending on external conditions, mainly temperature, pressure. Each of the compounds described in this section, namely AgI [61] and Bi_2_O_3_ [62], exhibit polymorphism, which can be present in several crystalline structures. These polymorphs can have different physical properties, such as electrical conductivity, important for applications.

### 3.1. Stabilization of α-AgI at Room Temperature

Silver iodide is a very versatile compound that can adopt several crystalline structures (e.g., α, β, γ). The most interesting of them, for both purely scientific reasons and applications, is α-AgI. This is a high-temperature phase, stable above 147 °C, i.e., above the temperature of the β-α phase transition. The best-known characteristic of this phase is its extremely high ionic (Ag^+^ ions) conductivity of ca 1 Scm^−1^ (at 150 °C), which is comparable to that of the best liquid electrolytes. Such high conductivity is a direct consequence of the specific crystalline structure of this phase. In the regular bcc unit cell of α-AgI, there are only two Ag^+^ ions which can occupy 42 different weakly bound, mostly tetrahedrally coordinated, crystallographic sites. As a result, silver ions can “almost freely” travel between these positions. The activation energy for Ag^+^ ion transport in α-AgI is very low (ca 0.05 eV). It should be noted that bulk AgI at room temperature is present as β-AgI a stable phase of the hexagonal (wurtzite) structure and low ionic conductivity [63]. Upon heating, there is a phase transition at 147 °C, at which the structure changes from β to α-AgI (regular fcc structure) with quasi-molten cationic (Ag^+^) sublattice (with activation energy lower than 0.1 eV). Its ionic conductivity is very high ca 1 Scm^−1^ at 150 °C. On the cooling the reverse phase transition takes place at lower temperatures, but one observes a temperature hysteresis. Moreover, the β-phase coexists with the metastable γ-phase (sphalerite structure). 

High ionic conductivity of α-AgI has been considered attractive for many applications. In particular, this structure has seemed to be very prospective as a solid electrolyte in silver electrochemical power sources. These advantageous features of α-AgI have prompted world-wide research on stabilizing α-AgI down to low temperatures, with a hope of preserving its high conductivity also at room temperature. The first successful results of these efforts were published in 1991 in Nature by Tatsumisago et al. [26]. Their approach consisted in fast-quenching of AgI–Ag_2_O–B_2_O_3_ melts very rich in AgI (even above 90% mole of AgI). They found out that a standard quenching (cooling rate of ca 10^3^ Ks^−1^) of the melts extremely rich in AgI, resulted in solid composites of a glassy phase and β-AgI phase. However, the same melts, when quenched at much higher rates (ca 10^5^ Ks^−1^) using the twin-roller technique, solidified as glassy-crystalline composites containing some fraction of small crystallites of α-AgI. The conductivity of that material at room temperature was very high (ca 0.1 Scm^−1^) and the activation energy was low (0.15 eV in the 20–140 °C. The authors attributed such advantageous electrical parameters of the synthesized glass-ceramic material to highly dispersed, discrete particles of frozen-in α-AgI. The authors’ further work in this direction showed that the stabilization of α-AgI in this way was reproducible [64]. It was confirmed that the high rate of melt-quenching by twin rollers technique (ca 10^5^ Ks^−1^) was crucial for obtaining glass-ceramic composites with α-AgI crystallites of 20–40 nm in size. In the conclusion section of ref. [64], it was stated that the stabilization of nanosize crystallites of α-AgI in silver borate glasses was possible due to large stresses at the crystallites-glass matrices, induced during the fast quenching.

Other researchers have also successfully extended downwards the stability range of α-AgI. Funke, Nowiński et al. [65] showed that the appropriate annealing of AgI-rich glasses (obtained by rapid quenching) of the composition 0.78AgI·0.165Ag_2_O·0.055B_2_O_3_ led to their nanocrystallization. The predominant crystalline phase was α-AgI. By modelling the ionic transport, it was concluded that the α-AgI phase stabilized at low temperature does not conduct like its high-temperature counterpart. Instead of a liquid-like, almost free motion of Ag^+^ ions prevailing at high temperatures (i.e., above 147 °C), the Ag^+^ transport in low-temperature stabilized α-AgI crystallites occurred by a solid state hopping of Ag^+^ [65]. Therefore, in that case the ionic conductivity of the stabilized α-AgI at room temperature was substantially lower than the estimate obtained by the extrapolation of the conductivity of the high-temperature α-AgI phase down to room temperature.

Another approach to stabilize α-AgI down to room temperature was studied by Makiura et al. [66]. They stabilized the α-AgI phase by coating very fine AgI nanoparticles (ca 11 nm) by thin layers of PVP polymer and subjecting them to a programmed thermal treatment. As a result, they obtained small particles of α-AgI. The ionic conductivity of these particles at room temperature was ca 10^−2^ Scm^−1^, which was much higher than the conductivity of the β-AgI present in polycrystalline materials at room temperature (10^−7^ Scm^−1^) [66]. The stabilization of the α-phase was also achieved by forming AgI nanowires inside an anodic aluminum oxide (AAO) membrane and treating them thermally. The final product contained α-AgI nanowires (60 nm) stable down to 80 °C [67]. It should be noted that the electrical conductivity of this heterogeneous material was lower than that of bulk α-AgI but substantially higher than that of β-AgI, and was stable in time. 

### 3.2. Stabilization of δ-Bi_2_O_3_ at Room Temperature

Bismuth sesquioxide (Bi_2_O_3_) is a very interesting compound which can adopt several different structures. Two of them (α and δ) are stable in their respective temperature ranges: 20–730 °C and 730–825 °C, respectively. Other forms, denoted as β, γ, ε, and ω, are metastable. However, all these structures are related to each other, and significantly associated with high-temperature δ-Bi_2_O_3_ [62]. From all these phases, the best known and most intensively studied is the δ-phase.

δ-Bi_2_O_3_ is a superionic oxide conductor (O^2−^ ions) whose ionic conductivity of ca 1 Scm^−1^ at 750°C is the highest among all known oxide ion conductors in that temperature range [68]. This conductivity is comparable to that of yttria stabilized zirconia (YSZ) ceramics at much higher temperature, namely at ca. 1000 °C. At present, YSZ ceramics with its many outstanding physicochemical properties and very well-developed technology at the industrial scale, still remains the main solid electrolyte for solid oxide fuel cells (SOFCs). A major problem with SOFCs using YSZ electrolytes is their very high operation temperature (ca 1000 °C), determined by the fact that the conductivity of YSZ attains an acceptable level 0.1–1 Scm^−1^ at ca 1000 °C only. Such a high operation temperature poses a lot of technical problems, requires special alloys or ceramics for construction elements, needs special sealing materials etc. Additionally, it leads to an accelerated degradation of the fuel cells. The replacement of YSZ by Bi_2_O_3_ could, in principle, lead to a substantial lowering of the operation temperature down to below ca 700–800 °C. This would start a breakthrough in SOFCs technologies, simplify manufacturing, lower the costs and, as a result, would lead to a substantial increase of the SOFCs’ production and usage. At present, the prospects of using Bi_2_O_3_ as a possible solid electrolyte for SOFCs are uncertain, because of several of its shortcomings. The first of them is a narrow temperature stability range (730–825 °C) of the high-conducting δ-phase, exhibiting fluorite structure. Outside that range the compound either undergoes melting (above 825 °C) or transforms into much less conductive metastable β- and γ phases [69] (below 730 °C). 

There have been many attempts to extend the temperature stability range of the δ-Bi_2_O_3_-like phase to lower temperatures (i.e., lower than 730 °C). Most of them involved solid solutions of Bi_2_O_3_ with selected oxides, like Y_2_O_3_ [70,71], WO_2_ [72] or others (e.g., [73,74]). Though these solid solutions retained the fluorite structure to temperatures much lower than 730°C, their electrical conductivity was much lower than that of pure δ-Bi_2_O_3_. Moreover, the pure delta phase of Bi_2_O_3_ is not retained in such solid solutions. Those and earlier studies have also shown that, depending on the ionic radius of the substituting elements, one can stabilize either a δ-like fluorite structure (ionic radii comparable to that of Bi^3+^ in octahedral coordination, i.e., 1.03 Å, e.g., Y^3+^ 0.90 Å [75]) or a γ-like one (when their ionic radii are much smaller, e.g., Si^4+^ 0.40 Å or Al^3+^ 0.54 Å [75]). 

A different successful approach to extend the stabilization range of the δ-Bi_2_O_3_ below 730 °C consisted in growing thin films of Bi_2_O_3_ on selected substrates by many techniques (e.g., [76,77,78]). However, that method was limited to thin films only, did not follow a glass-ceramic route, and is therefore out of the scope of this review.

A thermal nanocrystallization processing was tested to obtain Bi_2_O_3_ glass-ceramics which could be a possible alternative for typical solid-state syntheses [79]. Firstly, the Bi_2_O_3_ glasses were prepared by a melt-quenching from the molten Bi_2_O_3_. Their amorphousness was confirmed by XRD patterns which consisted of a wide halo with no Bragg peaks. The solidification of Bi_2_O_3_ melt as glass was surprising, because this pure compound does not belong to effective glass-formers (e.g., [80]). The elemental analysis of the final material carried out by energy-dispersive X-ray spectroscopy (EDS) revealed that it contained a small concentration of Si and Al impurities, coming from the ceramic crucibles used in syntheses. The oxides of these impurities, known as glass formers, could have partly facilitated the amorphization of Bi_2_O_3_. In the next step, the Bi_2_O_3_ glasses were thermally treated to induce their crystallization. That process was monitored by several complementary experimental methods probing the evolution of structure of newly appearing crystalline phases vs. temperature (by temperature-dependent XRD), microstructure (by SEM and HR-TEM) and the electrical (ionic) conductivity (by the impedance spectroscopy–IS). 

**Figure 14:**
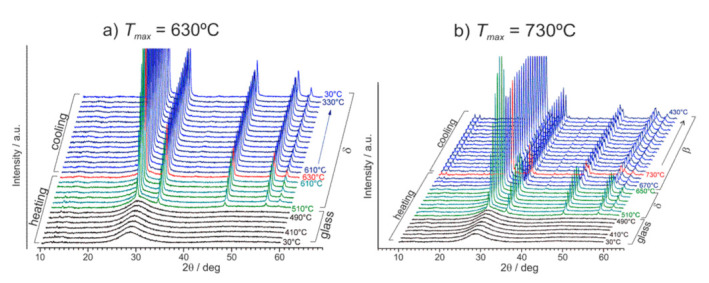
XRD patterns of initially amorphous Bi_2_O_3_ samples at series of temperatures on heating and cooling stages up to: (**a**) 630 °C and (**b**) 730 °C. Temperature intervals are 20 °C on both heating and cooling ramps. (Reproduced with permission from ref. [79]. Copyright 2018, Elsevier).

**Figure 15:**
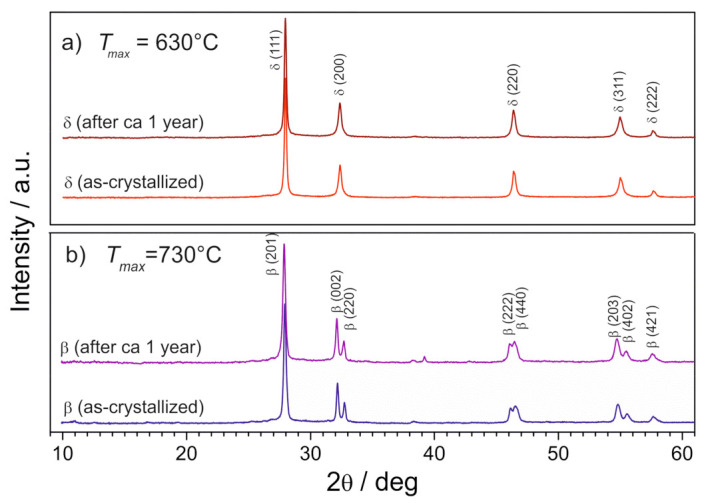
Indexed XRD patterns of Bi_2_O_3_ glass-ceramics samples heated up to: (**a**) 630 °C and (**b**) 730 °C and cooled down to room temperature. Patterns were collected for the same samples just after their crystallization and after one year of storage at room temperature. (Reproduced with permission from ref. [79]. Copyright 2018, Elsevier).

**Figure 16:**
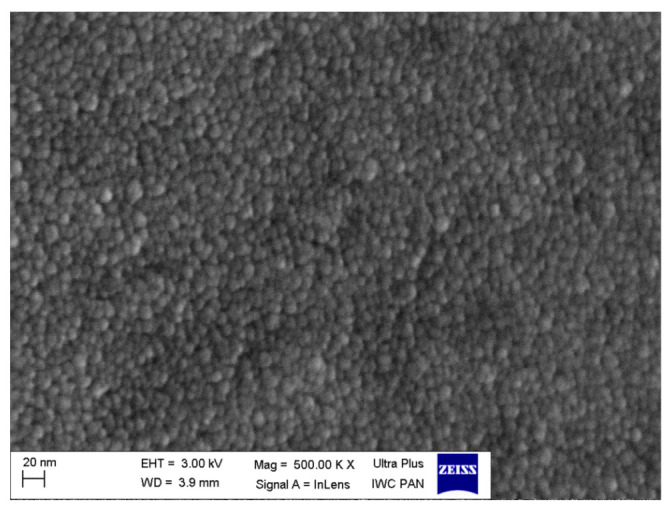
SEM image of a glass-ceramic Bi_2_O_3_ sample (at room temperature) obtained by a melt-quenching, followed by heating up to 630 °C. Microstructure consists mostly of 10–20 nm crystallites with the δ-Bi_2_O_3_ structure, as evidenced by XRD patterns (T.K. Pietrzak, unpublished results).

It was observed that the initially amorphous material started to crystallize at ca 510 °C (Figure 14a). The crystalline phase was identified as a fluorite-like δ-Bi_2_O_3_ (ICDD PDF 27–0052). Further temperature increase, until 630 °C, did not change the positions of the peaks. It led only to their slight narrowing and to an increase of their intensities. Those results mean that in the 510–630 °C temperature range, the residual glassy Bi_2_O_3_ matrix contains crystallites of δ-Bi_2_O_3_ phase. When the temperature range was extended up to 730 °C (Figure 14b), then at ca 650 °C the diffraction pattern changed, indicating a phase transition. The new phase was identified as β-Bi_2_O_3_ (ICDD PDF 77–5541). On cooling, both phases (δ-Bi_2_O_3_ in Figure 14a and β-Bi_2_O_3_ in Figure 14b) were stable down to room temperature. These observations are surprising because the δ−phase, in bulk form, is stable in the 730–825 °C temperature range only. An additional check of the time stability of the Bi_2_O_3_ glass-ceramics was made a year after the initial XRD measurements. The XRD patterns of both materials, annealed up to 630 °C and 730 °C are identical with the original ones (Figure 15). This means that both crystalline phases (δ and β) present in small grains embedded inside a glass matrix are stable at room temperature for at least a year. In other words, the high-temperature δ-Bi_2_O_3_ can be stabilized down to room temperature in nanocrystallites confined in the glass. This stabilization effect could be attributed to the influence of the nanocrystallites’ surfaces and the impact of the surrounding glassy phase on the crystallites’ interiors. These factors can cause local stresses and affect the structure inside the crystallites, determining the most favorable Bi_2_O_3_ crystalline structure under given local conditions. The microstructure of these materials annealed at 630 °C consists of a large number of small, mostly 10–20 nm crystalline grains (Figure 16). Our lab independently showed that a similar microstructure could be obtained in a very rapid single-step quenching (by a twin-roller technique) of molten Bi_2_O_3_.

**Figure 17:**
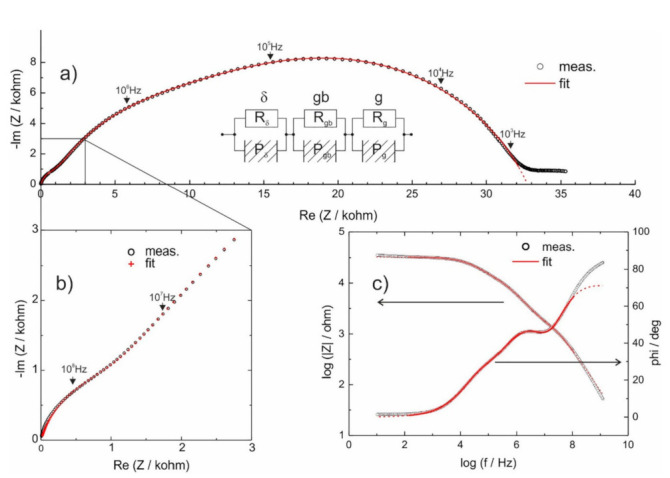
(**a**) Impedance spectrum of a sample heat-treated at 530 °C, measured in a frequency range 10 GHz–10 Hz (fitted in the 200 MHz–200 Hz range). Open circles denote experimental points, the solid line corresponds to the numerical fit using the equivalent circuit shown in the inset. (**b**) A magnified high-frequency part of the spectrum a) (marked by a solid line box). Open circles denote experimental points and “+” correspond to fits. (**c**) Impedance spectrum in the Bode representation. (Reproduced with permission from ref. [79]. Copyright 2018, Elsevier).

The electrical conductivity of these materials was measured by impedance spectroscopy in two frequency ranges: a standard one (0.1 Hz–20 MHz [81]) and a broad-band one (10 Hz–10 GHz [82,83]). The obtained spectra were fitted and analyzed using the equivalent circuit approach. It should be noted that the impedance spectra measured in both frequency ranges led to similar overall conductivity results at given temperatures. However, only by using the broad-band spectra, it was possible, through the numerical fitting, to extract information on the conductivity of the δ-Bi_2_O_3_ crystallites [79]. An impedance spectrum of a Bi_2_O_3_ glass-ceramics measured at 530 °C, together with the electric equivalent circuit and the numerical fit, are shown in Figure 17a (Nyquist representation) and Figure 17b,c (Bode representation). The three (R-CPE) loops, where CPE stands for constant phase element, in the equivalent circuit shown in the inset, are attributed to the glassy phase (R_g_), grain-boundaries (R_gb_), and crystallites of the δ-Bi_2_O_3_ (R_δ_), respectively. 

The total electric conductivity of the Bi_2_O_3_ increased with temperature according to the Arrhenius formula with the activation energy of 1.24 eV on heating. On cooling, the temperature dependence of the conductivity was very similar and the activation energy was 1.30 eV [79]. This effect stems from the fact that at least three components determine the total conductivity: the glassy phase, grain boundaries, and crystallites of the δ-phase. 

**Figure 18:**
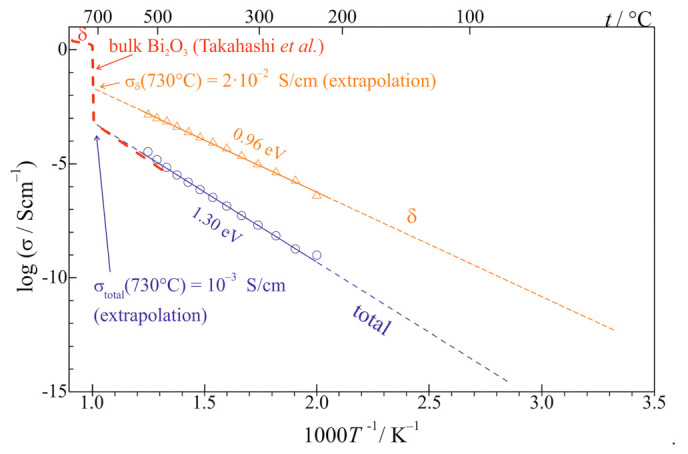
Temperature dependence of conductivity of a glass-ceramic Bi_2_O_3_ sample: total conductivity (circles), conductivity of the δ-like phase determined from analyses of the impedance spectra (triangles) together with the respective fits (straight lines). The data for the high temperature δ phase of bulk Bi_2_O_3_ (red dashed line) (after Takahashi et al. [84]) are included for comparison. (Reproduced with permission from ref. [79]. Copyright 2018, Elsevier).

The summary of the conductivities of the nanocrystallized Bi_2_O_3_ is presented in Figure 18. It shows that the total conductivity is moderate and follows the Arrhenius formula with the activation energy of 1.30 eV. The conductivity of the d-phase is approximately two orders of magnitude higher, but still lower than expected from the extension of the high-temperature dependence reported by Takahashi et al. for bulk δ−Bi_2_O_3_ (Figure 18) [84]. The activation energy, which in Takahashi’s work was about 0.4–0.5 eV, in our case is different (higher), namely 0.96 eV. A similar discrepancy has also been reported in the thin films of δ-Bi_2_O_3_ stabilized at low temperatures where the activation energy at low temperatures was close to 1 eV. Both these conductivities (total and δ) are compared to the conductivity of the bulk crystalline Bi_2_O_3_ (a dashed line) showing a step-like increase at ca 730 °C which corresponds to a phase transition of Bi_2_O_3_ to its highly conducting δ-phase. Further studies, necessary to explain the δ-phase stabilization down to room temperature in Bi_2_O_3_ glass-ceramics, are in progress. 

## 4. Conclusions

Thermal nanocrystallization of oxide glasses is an effective method to synthesize glass-ceramic materials providing enhanced electrical conductivity by the factor from approximately 1000 up to over 10^7^ (e.g., electronically conducting vanadate-phosphate and olivine-like nanocomposites) or by a factor of ca. 4 (e.g., in ionically conducting Ag-rich silver glasses). The method also offers a possibility of stabilization of highly conducting high-temperature phases down to room temperature (α-AgI crystallites embedded in silver borate glasses or grains of δ-phase confined in Bi_2_O_3_ glasses). 

What all those nanostructured materials have in common is their origin (the glassy state) and the presence of nanocrystallites (5–100 nm) confined in a residual glassy matrix. Until now, the studies on phenomena observed in such heterogeneous and disordered systems have not been very intensive, especially in comparison to the fast developing field of thin films deposition nanotechnology. Another common feature of glass-ceramics described in this review is their potential application in devices for energy storage or conversion as solid electrolytes (ionic conduction) or electrodes (electronic conduction). 

Given the practical advantages of those glass-ceramics and still unsatisfactory state of our knowledge, it would be very advisable to clarify in detail the mechanisms of electric charge transport in those heterogeneous materials and their correlation with their microstructure. From the practical point of view, such materials, prepared by inexpensive and scalable processing, can be an important alternative to worldwide studied solid electrodes and electrolytes prepared by other, more cost-intensive routes. Theis review is focused on oxide systems, however it is known that similar effects and phenomena have also been observed in other glassy systems.

## Data Availability

Not applicable.
